# Role of Renal Nerves in the Treatment of Renovascular Hypertensive Rats with *L*-Arginine

**DOI:** 10.1155/2014/735627

**Published:** 2014-09-30

**Authors:** Sonia Alves Gouvea, Renata V. Tiradentes, Cintia H. Santuzzi, Vinícius Mengal, Henrique de A. Futuro Neto, Nyam F. Silva, Gláucia R. Abreu

**Affiliations:** ^1^Department of Physiological Sciences, Health Sciences Center, Federal University of Espírito Santo, Avenida Marechal Campos 1456, 29040-577 Vitória, ES, Brazil; ^2^Department of Morphology, Health Sciences Center, Federal University of Espirito Santo, Vitoria, ES, Brazil; ^3^Faculdade Brasileira Medical School, Vitoria, ES, Brazil; ^4^EMESCAM Medical School, Vitoria, ES, Brazil

## Abstract

The purpose was to determine the role of renal nerves in mediating the effects of antihypertensive treatment with *L*-arginine in a renovascular hypertension model. The 2K1C (two-kidney one-clip model) hypertensive rats were submitted to bilateral surgical-pharmacological renal denervation. The animals were subdivided into six experimental groups: normotensive control rats (SHAM), 2K1C rats, 2K1C rats treated with *L*-arginine (2K1C + *L*-arg), denervated normotensive (DN) rats, denervated 2K1C (2K1C + DN) rats, and denervated 2K1C + *L*-arg (2K1C + DN + *L*-arg) rats. Arterial blood pressure, water intake, urine volume, and sodium excretion were measured. The 2K1C rats exhibited an increase in the mean arterial pressure (MAP) (from 106 ± 3 to 183 ± 5.8 mmHg, *P* < 0.01), whereas *L*-arg treatment induced a reduction in the MAP (143 ± 3.4 mmHg) without lowering it to the control level. Renal nerve denervation reduced the MAP to normotensive levels in 2K1C rats with or without chronic *L*-arg treatment. *L*-arg and denervation induced increases in water intake and urine volume, and *L*-arg caused a significant natriuretic effect. Our results suggest that renal sympathetic activity participates in the genesis and the maintenance of the hypertension and also demonstrate that treatment with *L*-arg alone is incapable of normalizing the MAP and that the effect of such treatment is not additive with the effect of kidney denervation.

## 1. Introduction

The role of sympathetic innervation in renovascular hypertension has long been a topic of intensive investigation [1]. Several lines of evidence suggest that increased activity in afferents from diseased kidneys can enhance sympathetic nerve activity, facilitate the progression of blood pressure elevation, and promote hypertension-related target organ damage [2–5]. Therefore, renal sympathetic nervous activity (RSNA) has an important role in the control of renal blood flow (RBF) and the glomerular filtration rate (GFR) and can thereby influence the extracellular volume and the arterial blood pressure [6, 7]. The removal of neural input through renal denervation leads to the inhibition of tubular transport (diuresis and natriuresis) and, under some circumstances, to a decrease in renin secretion and angiotensin II (Ang II) formation [8]. Nevertheless, changes in efferent renal nerve activity independently of reflex afferent activity are essential in the onset and maintenance of hypertension in humans and in animal models [9–12]. Thus, both researchers and clinicians consider sympathetic denervation to be an important goal of therapeutic interventions aimed at reducing elevated blood pressures and, more generally, at correcting or slowing down the pathologic processes responsible for disease complications. Examples of pathologic processes secondary to intense hypertension include heart failure, renal insufficiency, and myocardial infarction [13–16].

The two-kidney one-clip (2K1C) renovascular hypertension model is dependent on Ang II, and various studies have demonstrated elevated circulating levels of Ang II with high Ang II concentrations in the cortical tissue of the clipped and nonclipped kidneys [17]. It is well known that the production of Ang II is dependent on the renin release that is stimulated by increased sympathetic activity [18–20]. In this model of hypertension, the inhibition of nitric oxide (NO) synthesis results in an exaggerated increase in the systemic blood pressure and in a decrease in RBF in the nonclipped contralateral kidney [21]. Several studies have also demonstrated an important effect of* L*-arginine (*L*-arg) on different hypertension models [3, 4, 22, 23]. Therefore, the administration of exogenous* L*-arg could, via an increase in the production of NO, restore endothelial dysfunction and reduce blood pressure in individuals with renovascular hypertension [24].

The aim of the present study was to investigate the relationship between renal denervation and* L*-arg treatment and the contributions of these treatments to the regulation of sodium and water excretion and of MAP in a model of renovascular hypertension (2K1C).

## 2. Material and Methods

Male normotensive Wistar rats weighing 150–170 g were obtained from the Federal University of Espirito Santo animal house. The animals were housed in a temperature- and humidity-controlled room (25°C) with a 12 h light cycle. Standard food pellets and tap water were provided* ad libitum*.

The project was approved by the Institutional Ethics Committee for Animal Research (Ethics Committee for the Use of Animals, UFES, Protocol 044/2010), and all experiments were conducted in accordance with the Guide for the Care and Use of Laboratory Animals published by the US National Institutes of Health (NIH Publication 85-23, revised 1996).

The animals were randomly allocated into six experimental groups (*N* = 8 per group): normotensive control (SHAM) rats, two-kidney one-clip (2K1C) rats, two-kidney one-clip rats treated with* L*-arginine (2K1C +* L*-arg), denervated normotensive (DN) rats, denervated two-kidney one-clip (2K1C + DN) rats, and denervated two-kidney one-clip rats treated with* L*-arginine (2K1C + DN +* L*-arg).

### 2.1. Surgical Procedures and Experimental Protocol

Rats underwent bilateral renal denervation to eliminate direct and reflex influences on renal hemodynamics and excretory function. Under sodium thiopental (50 mg/kg,* i*.*p*.) anesthesia, the left kidney was exposed via a flank incision. The adventitia surrounding the right renal artery and vein was stripped, and all visible renal nerves were cut under a surgical microscope (D.F. Vasconcellos 902, São Paulo, SP, Brazil). The vessels were then treated with 95% alcohol containing 10% phenol. This renal denervation procedure prevents the renal vasoconstrictor response to suprarenal lumbar sympathetic nerve stimulation, prevents the antinatriuretic response to environmental stress, and reduces the renal tissue norepinephrine concentration to <5% of the control concentration for up to 15 days after denervation [25].

After renal denervation, the flank incision was sutured, and the procedure was repeated on the opposite side to denervate the right kidney. Then, a silver clip (ID, 0.2 mm) was placed around the left renal artery. Denervated normotensive rats underwent a similar procedure with manipulation of the right and left renal arteries but without permanent placement of the clip [22, 25]. Immediately after the surgery, the animals received an appropriate dose of antibiotic (2.5% enrofloxacin, 0.1 mL per rat,* i*.*m*.).

**Figure 1:**
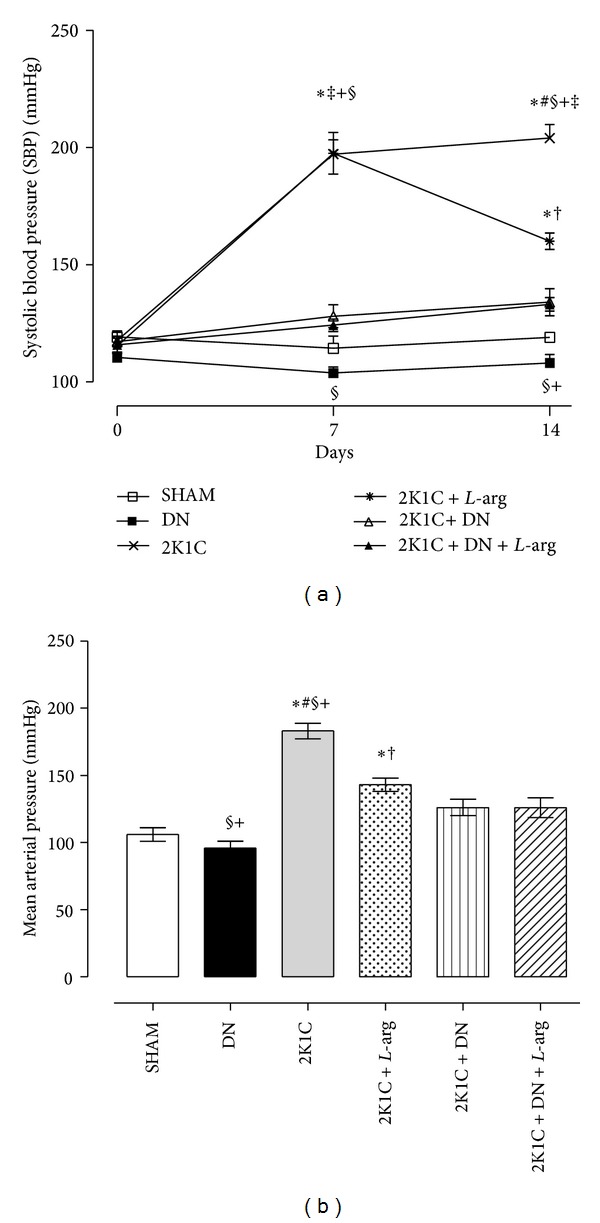
(a) Systolic blood pressure data measured by tail cuff in renovascular hypertensive rats (time 0: baseline before surgery, 7th day after surgery and/or denervation, and 14th day after surgery and/or denervation and* L*-arginine treatment). (b) Mean arterial pressures at the end of experiment in conscious rats for all experimental groups (SHAM: normotensive control; 2K1C: two-kidney one-clip; 2K1C +* L*-arg: two-kidney one-clip treated with* L*-arginine; DN: denervated normotensive; 2K1C + DN: denervated two-kidney one-clip; and 2K1C + DN +* L*-arg: denervated two-kidney one-clip treated with* L*-arginine). The results are expressed as mean ± SEM. *P < 0.05 versus SHAM; ^†^ *P* < 0.05 versus 2K1C; ^‡^ *P* < 0.05 versus DN; ^#^ *P* < 0.05 versus 2K1C +* L*-arg; ^§^ *P* < 0.05 versus 2K1C + DN, and ^+^ *P* < 0.05 versus 2K1C + DN +* L*-arg (Tukey test).

Seven days after surgery, the animals were placed individually into metabolic cages, and the animals of the 2K1C +* L*-arg and 2K1C + DN +* L*-arg groups were treated with* L*-arginine (Sigma, St. Louis, MO, USA) at a dose of 10 mg/mL in the drinking water for 7 days. Water intake and the urinary volume were measured daily. The urinary sodium content was determined by flame photometry (Micronal B262, São Paulo, SP, Brazil) and expressed as mEq/day [22, 25]. The systolic blood pressure was measured by tail cuff plethysmography, to determine the time of onset of hypertension, and at the end of the experiments (Figure 1(a)). At the end of the initial experimental protocol (15 days), the animals were anesthetized with sodium thiopental (50 mg/kg,* i*.*p*.), and a catheter made of PE-50 tubing connected to PE-10 tubing was passed through the right femoral artery. The catheter was threaded through the blood vessels so that it exited at the back of the neck. After insertion, the catheter was flushed and filled with heparinized saline (40 U/mL). Twenty-four hours later, the MAP was measured in conscious, freely moving animals using a pressure transducer (model PT 300; Grass Instruments Div., Warwick, NY, USA) coupled to a bridge amplifier and a digitizer (Biopac, MP100, Santa Barbara, CA, USA). At the end of the experimental procedures, the rats were killed with an overdose of anesthetic, and the kidneys were removed and stored frozen (−22°C) for later measurement of the norepinephrine concentration. After the acute experiments, the kidneys were removed under anesthesia and stored frozen to measure the norepinephrine concentration by fluorometric assay (Hitachi F-2000).

### 2.2. Statistical Analysis

The results are presented as the mean ± SEM. Statistical significance was determined using one-way ANOVA for repeated measures, followed by Tukey's test with a level of significance set at *P* < 0.05.

## 3. Results

The systolic blood pressure (SBP) data were measured by tail cuff during the experimental study in all experimental groups (SHAM, DN, 2K1C, 2K1C +* L*-arg, 2K1C + DN, and 2K1C + DN +* L*-arg). The baseline SBP (time 0) was similar in the six experimental groups before surgery (SHAM: 119.2 ± 2.51 mmHg, *N* = 8; DN: 110.4 ± 2.30 mmHg, *N* = 8; 2K1C: 117.6 ± 3.6 mmHg, *N* = 8; 2K1C +* L*-arg: 115.4 ± 3.2 mmHg, *N* = 8; 2K1C + DN: 117.2 ± 2.26 mmHg, *N* = 8; and 2K1C + DN +* L*-arg: 115.8 ± 3.50 mmHg, *N* = 8) (Figure 1(a)). Seven days after surgery and denervation, the SBP increased in 2K1C and 2K1C +* L*-arg groups (2K1C: 197.1 ± 6.08 mmHg; 2K1C +* L*-arg: 197.5 ± 8.90 mmHg, *P* < 0.05) compared to SHAM, DN, 2K1C + DN, and 2K1C + DN +* L*-arg (SHAM: 114.4 ± 5.2 mmHg; DN: 103.8 ± 2.5 mmHg; 2K1C + DN: 128 ± 4.9 mmHg; 2K1C + DN +* L*-arg: 124.2 ± 2.7 mmHg). Additionally, DN group (103.8 ± 2.5 mmHg, *P* < 0.05) showed significant reduction in SBP compared to the 2K1C + DN group (128 ± 4.9 mmHg) (Figure 1(a)). On the fourteenth day after* L*-arg treatment, the 2K1C +* L*-arg group (160 ± 3.5 mmHg, *P* < 0.05) reduced the SBP compared to 2K1C group (204 ± 5.8 mmHg) and SBP remained elevated when compared to the SHAM group (119.1 ± 1.9 mmHg). On the other hand, the DN group (108.1 ± 3.6 mmHg, *P* < 0.05) had significantly lower SBP compared with 2K1C + DN and 2K1C + DN +* L*-arg groups (134 ± 5.8 mmHg; 133.1 ± 2.9 mmHg, resp.) (Figure 1(a)).

The MAP values obtained on the 15th day after surgery in conscious rats are shown in Figure 1(b). The 2K1C group (183 ± 5.8 mmHg, *P* < 0.05) maintained high MAP compared with all experimental groups (SHAM: 106 ± 3 mmHg; DN: 96 ± 1.8 mmHg; 2K1C +* L*-arg: 143 ± 3.4 mmHg; 2K1C + DN: 126 ± 6.1 mmHg; and 2K1C + DN +* L*-arg: 126 ± 7.3 mmHg). Similarly, 2K1C +* L*-arg (143 ± 3.4 mmHg) reduced MAP compared to 2K1C (183 ± 5.8 mmHg) and DN group (96 ± 1.8 mmHg) MBP was still reduced compared to 2K1C + DN (126 ± 6.1 mmHg) and 2K1C + DN +* L*-arg (126 ± 7.3 mmHg) groups.

**Figure 2:**
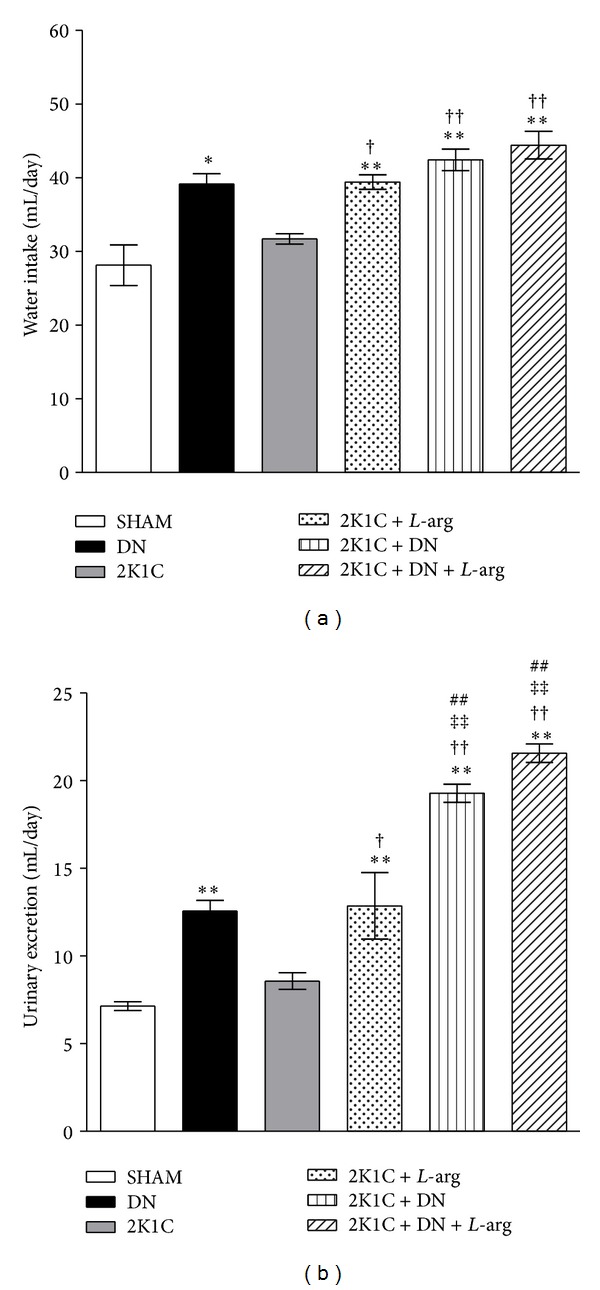
Water intake (a) and urinary excretion (b) after oral* L*-arginine (*L*-arg) administration to the SHAM, two-kidney one-clip, and denervated groups. The groups are as follows: SHAM: normotensive control; 2K1C: two-kidney one-clip; 2K1C +* L*-arg: two-kidney one-clip treated with* L*-arginine; DN: denervated normotensive; 2K1C + DN: denervated two-kidney one-clip; and 2K1C + DN +* L*-arg: denervated two-kidney one-clip treated with* L*-arginine. The data are shown for 7 days of* L*-arg administration. Data are reported as the means ± SEM. *P < 0.05 and **P < 0.01 compared with SHAM; ^†^ *P* < 0.05 and ^††^ *P* < 0.01 compared with 2K1C; ^‡‡^ *P* < 0.01 compared with DN; ^##^ *P* < 0.01 compared with 2K1C +* L*-arg (Tukey test).

The daily mean water intake and excretion (Figure 2) were significantly higher (*P* < 0.01) in the 2K1C +* L*-arg group (39.4 ± 1 and 12.8 ± 2 mL/day) than in the SHAM and 2K1C groups (28 ± 2.8 and 7.1 ± 0.2 mL/day; 31.7 ± 0.7 and 8.6 ± 0.55 mL/day, resp.). Additionally, in the denervated groups, water intake and excretion (39 ± 1.4 and 12.5 ± 0.4 mL/day in the DN group; 42.4 ± 1.5 and 19.2 ± 0.6 mL/day in the 2K1C + DN group; 44.4 ± 1.9 and 21.5 ± 0.8 mL/day in the 2K1C + DN +* L*-arg group) were significantly higher (*P* < 0.01) than in the SHAM rats (28 ± 2.8 and 7.1 ± 0.2 mL/day). In the denervated groups, there were no significant intergroup differences in water intake, but the urinary excretion levels of the 2K1C + DN and 2K1C + DN +* L*-arg groups were significantly higher than those of the DN group.

Additionally, the 2K1C + DN group had a significantly (*P* < 0.01) greater level of water intake (Figure 2) than the animals in the 2K1C and SHAM groups (42.4 ± 1.5 versus 31.7 ± 0.7; 28 ± 2.8 mL/day, resp.) and a greater level of urinary excretion than the SHAM, DN, 2K1C, and 2K1C +* L*-arg groups. This result was also observed for the 2K1C + DN +* L*-arg group (Figure 2).

**Figure 3:**
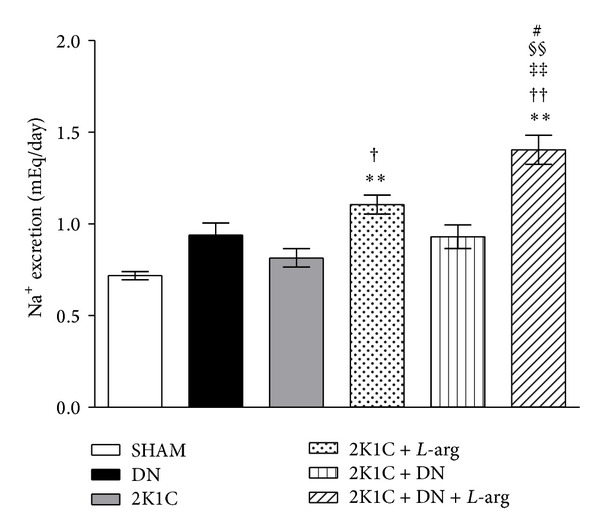
Urinary sodium excretion after oral* L*-arginine (*L*-arg) administration to the SHAM, two-kidney one-clip, and denervated groups. The groups are as follows: SHAM: normotensive control; 2K1C: two-kidney one-clip; 2K1C +* L*-arg: two-kidney one-clip treated with* L*-arginine; DN: denervated normotensive; 2K1C + DN: denervated two-kidney one-clip; and 2K1C + DN +* L*-arg: denervated two-kidney one-clip treated with* L*-arginine. The data are shown for 7 days of* L*-arg administration. Data are reported as the means ± SEM. **P < 0.01 compared with SHAM; ^††^ *P* < 0.01 compared with DN; ^†^ *P* < 0.05 and ^††^ *P* < 0.01 compared with 2K1C; ^#^ *P* < 0.05 compared with 2K1C +* L*-arg; *P* < 0.05 compared with 2K1C + DN (Tukey test).

The level of sodium excretion was significantly higher (*P* < 0.01) in the 2K1C +* L*-arg group than in the untreated and SHAM groups (1.1 ± 0.05, 0.8 ± 0.05, and 0.72 ± 0.02 mEq/day, resp.). In addition, the treatment associated with denervation (1.4 ± 0.08 mEq/day) further increased the level of sodium excretion relative to that of the other groups (0.94 ± 0.06 mEq/day in the DN group and 0.93 ± 0.06 mEq/day in the 2K1C + DN group) (Figure 3). The levels of potassium excretion were similar in all groups (data not shown).

The success of the renal denervation procedure was confirmed by the reduction of the concentration of renal tissue norepinephrine to undetectable levels in the renal-denervated groups, compared with 422 ± 25 ng/g wet kidney weight in the normotensive control group (SHAM-operated group).

## 4. Discussion

The present study was designed to assess the relative contribution of* L*-arg treatment to changes in sodium and water excretion and its influence on MAP when used in combination with renal denervation. Our data provide evidence that the administration of exogenous* L*-arg promoted increases in sodium and water excretion and a decrease in the MAP, although the MAP was not normalized. The abilities of* L*-arg to significantly increase sodium and water excretion and to lower the MAP were enhanced when combined with renal denervation. The effectiveness of the renal denervation procedure was confirmed by the reduction of the concentration of renal tissue norepinephrine. Therefore, the influence of renal nerves on kidney function was significantly removed [26]. A limitation of our study was not recording systemic sympathetic activity in the different groups. Nevertheless, recording of this parameter in denervated animals is not possible. On the other hand, it well established the relationship of renal sympathetic activity and systemic arterial pressure level in various hypertension models [27].

It has been postulated that the major factors in the development of hypertension are an increase in Ang II and endothelial dysfunction in 2K1C hypertension. It is suggested that a deficient production of endothelium derived NO results in diminished vasodilator tone, allowing vascular resistance to increase, and this contributes to the elevated blood pressure [28]. Moreover, overactivity of the sympathetic nervous system (SNS) has been implicated in the development and maintenance of essential and renovascular hypertension in humans [29–32]. The paraventricular nucleus (PVN) is an important component that regulates sympathetic outflow via projections to the intermediolateral column of the spinal cord and the Rostral ventrolateral medulla (RVLM). Within the PVN, Ang II enhances the sympathetic activity and blood pressure in 2K1C rats, whereas nitric oxide (NO) has been shown to have a sympathoinhibitory effect in the PVN [33, 34].

In addition, in the central nervous system, Ang II is able to increase sympathetic vasomotor tone and, therefore, increase blood pressure. This effect partially explains the involvement of Ang II in the pathogenesis of many experimental models of hypertension, including renovascular hypertension [3, 25]. The role of sympathetic activation in the 2K1C model has long been a topic of intensive investigation [35]. The renal sympathetic nerves have been identified as major contributors to the complex pathophysiology of hypertension in both experimental models and humans [36]. A modest increase in the circulating concentration of Ang II, which acts on the central nervous system via AT_1_ receptors, might contribute to NADPH activation, which leads to an increase in the local production of reactive oxygen species. Increased reactive oxygen species production causes sympathoexcitation and arterial hypertension, and the increase in oxidative stress in the brain, particularly in neurons involved in cardiovascular regulation and in the stenotic kidney, may play a major role in maintaining a high arterial pressure and sympathetic drive under conditions of renovascular hypertension [23].

One of the complications of high blood pressure is the deterioration in renal function, which may lead to an overt renal insufficiency state. In this case, sympathetic activity appears to be involved in the pathogenesis of renal disease, given that sympathetic activation is detectable in the initial forms of this pathologic condition, when the estimated GFR is only mildly impaired [37].

It is known that the modulation of renal function by RSNA is pivotal in regulating the extracellular fluid volume. Increases in efferent RSNA reduce RBF and urinary sodium excretion by activating *α*1-adrenoceptors [37]. Burke et al. demonstrated that the kidney function appears to be well preserved in animals that have had one kidney clipped and the blood pressure normalized by sympathetic inhibition [38]. Franquini et al. [39] demonstrated that renal denervation induced an increase in urine volume and sodium excretion. In contrast, DiBona and Sawin [26] showed that renal denervation prevents the renal vasoconstrictor response and antinatriuresis. These data are consistent with the results of our study, demonstrating that the removal of renal sympathetic innervation results in greater water excretion and, thus, an increase in water intake. The presence of a precursor of NO alone increased water and sodium excretion, and when associated with renal denervation, it induced an even greater water and sodium excretion. Previous studies in our laboratory demonstrated that, in the context of renovascular hypertension,* L*-arg treatment decreases the arterial pressure not only because of the already known vasodilator effects of NO formation but also because there is an increase in the renal excretion of water and sodium [40].

## 5. Conclusions

Our results demonstrate that renal efferent and afferent activity play a role in the induction and maintenance of renovascular hypertension. Treatment with* L*-arg demonstrates that NO participates in this process; nevertheless, its role is not crucial, as demonstrated by its inability to reverse the hypertensive state and return the blood pressure to the control level. It was also observed that these two procedures, that is, renal denervation and* L*-arg treatment, were not additive in nature. Our results highlight the participation of renal sympathetic nerve activity, as one of the casual agents, responsible for the acute induction and maintenance of two-kidney one-clip renovascular hypertension.
